# Hypothyroidism and hyperthyroidism related to gynecologic cancers: a nationwide population-based cohort study

**DOI:** 10.1038/s41598-023-50439-z

**Published:** 2024-01-22

**Authors:** John Hang Leung, Shyh-Yau Wang, Henry W. C. Leung, Teng-Shun Yu, Agnes L. F. Chan

**Affiliations:** 1 Department of Obstetrics and Gynecology, Ditmanson Medical Foundation Chia-Yi Christian Hospital, No. 539, Zhongxiao Road, East Dist., Chiayi, 60002 Taiwan; 2 Department of Radiology, An-Nan Hospital, China Medical University, No. 66, Sec. 2, Changhe Rd., Annan Dist., Tainan, Taiwan; 3 Department of Radiation Oncology, An-Nan Hospital, China Medical University, No. 66, Sec. 2, Changhe Rd., Annan Dist., Tainan, Taiwan; 4 Management Office for Health Data, Clinical Trial Research Center, China Medical University Hospital, No. 2, Yude Road, North District, Taichung, 40447 Taiwan; 5 Department of Pharmacy, An-Nan Hospital, China Medical University, No. 66, Sec. 2, Changhe Rd., Annan Dist., Tainan, 709 Taiwan

**Keywords:** Cancer, Immunology, Medical research, Oncology

## Abstract

The purpose of this study was to assess the risks of hyperthyroidism and hypothyroidism related to gynecological cancers. Population-based retrospective cohort study. We conducted a cohort study using the Taiwan National Health Insurance Research Database to explore hyperthyroidism and hypothyroidism associated with site-specific gynecologic cancers in women from January 1, 2000 to December 31, 2018. The examined gynecologic cancers included endometrial (EC), uterine corpus cancer (UC), and ovarian cancer (OC). The incidence and hazard ratios were quantified using Cox proportional hazards models. The incidence of developing gynecological (Gyn) cancers in the hyperthyroid and hypothyroid women was 0.29 and 0.44 per 1000 person-years, which was 0.86 fold lower and 1.13 fold higher than that in the comparison cohort (p < 0.001). Compared with patients aged 20–40 years, patients in older age groups had a lower and higher risk of developing Gyn cancers (for hyperthyroid, 40–65 years: adjusted hazard ratio (aHR) = 0.82; > 65 years: aHR = 0.94; for hypothyroid, adjusted hazard ratio (aHR) = 1.26; > 65 years: aHR = 1.38). Compared with the non-hypothyroid women and non-hyperthyroid women beyond 6 years of follow-up, hypothyroid and hyperthyroid women showed decreased risk of Gyn cancers. Medication treatment for hyperthyroid and hypothyroid disease did not showed significant association in subgroup analyses (aHR = 0.99 and 0.80, respectively). Our results show that women with hyperthyroidism have a significantly reduced risk of gynecological cancers, whereas women with hypothyroidism have a slightly increased risk of gynecological cancers suggesting an association between thyroid function level and risk of gynecological cancers.

## Introduction

Gynecological cancers are a major public health problem worldwide, with an estimated 1.3 million new cases diagnosed and over 6.7 million deaths in 2020^1^. In Taiwan, the three highest incidence rates in 2019 were endometrial cancer (corpus uteri) ovarian cancer and cervix uteri. The crude incidence rate in 2020 were 25.51 per 100,000 (endometrial cancer), 15.34 per 100,000 (ovarian cancer) and 12.08 per 100,000 (cervix uteri cancer)^2^. The incidence and mortality rates are continuously increasing and tend to be younger. Therefore Taiwan's National Health Service recommends that gynecological cancer cases should be detected early and selected appropriate cancer treatment strategies to prolong survival^2^.

Endometrial cancer (EC) is one of the most common gynecological cancer and is highly related to the endocrine system. Epidemiological studies reported that risk factors for EC may relate to metabolic syndrome, obesity, diabetes, hypertension, younger age at menarche, late age at menopause, infertility, nulliparity, age at birth of first child, long-term use of unopposed estrogens during hormone replacement therapy, tamoxifen use, polycystic ovary syndrome, and progression from atypical hyperplasia to cancer^3^. Epidemiological studies reported that patients with EC presented higher serum TSH levels^4–8^. Hypothyroidism might influence the development of EC by indirectly interfering with the risk factors or by directly acting on thyroid-related receptors and influencing the disease in other dependent mechanism^9^.

Ovarian cancer is hormone-dependent, and epidemiological evidence links hyperthyroidism, inflammation of the ovarian surface, and increased risk of ovarian cancer^10,11^. A population-based case–control study indicated that co-occurrence of thyroid dysfunction nearly doubles the risk of ovarian cancer^10^. Additionally, experimental studies have shown that primary ovarian surface epithelial cells may develop ovarian cancer and TRs are strongly expressed at the mRNA and protein levels^11,12^. They also found that the stimulation of ovarian surface epithelial cells with thyroid hormone (T3) induced an inflammatory gene-expression profile and up-regulated estrogen receptor alpha and matrix metalloproteinase^13^. Over the past decade, some studies have shown that increased inflammation and inflammatory mediators are associated with increased risk of EOC and decreased survival of patients with EOC, promoting the development of ovarian tumors^14^.

Thyroid hormones are important regulators of cellular processes associated with differentiation, metabolism, apoptosis and growth^15,16^. Their known mechanism of action depends on their ability binding to thyroid hormone receptors (TRs). In recent decades, many researchers have suggested that the mechanism by which thyroid hormones exert growth-promoting effects may be mediated by phosphatidylinositol-3-kinase and MAPK, and involve stimulation of angiogenesis through αvβ3 integrin binding^17–19^. A recently published comprehensive review of preclinical and clinical studies indicated that substantial evidence suggests that preclinical in vitro research suggest that the role of thyroid hormones in cancer is mediated by complex genomic and non-genomic signaling pathways and is highly dependent on cell type and molecular environment^20^. In vivo studies have shown that TH has broad effects on cancer development and progression through changes in local intracellular TH concentrations, which contribute to cancer cell proliferation and thereby stimulate tumor growth, while the extracellular hypothyroid environment may support cancer progression by attenuating the immune response. Conclusively, some evidence strongly suggests that non-genomic T4 actions can trigger cancerous proliferation, and that interference with T4-avb3 integrin binding, which could provide effective therapeutic options for patients. However, the result of clinical studies on the association of thyroid hormone and cancer is conflicting. Most studies indicated that clinical hyperthyroidism increases the risk of several solid malignancies, while hypothyroidism may reduce aggressiveness or delay cancer onset^21^. In contrast, hypothyroidism was associated with increased aggressiveness of colorectal and liver cancers, with comparable effects on cancer risk^22^.

Given that thyroid disease and altered thyroid hormone expression can affect ovulation, endometrial physiology, and estrogen levels, further exploration of possible potential relationship with gynecologic cancer risk is warranted. We therefore aimed to investigate the possible relationships of hypothyroidism and hyperthyroidism with the risk of gynecological cancers.

## Methods

### Data source

This was a population-based cohort study. We analyzed data retrieved from the catastrophic illness patient dataset (HV) of the Taiwan National Health Insurance Research Database (NHIRD) from 1 January 2000 to 31 December 2018^23–25^. The HV dataset is one of the subset databases in the NHIRD. Cancers are defined as a catastrophic illness by the government. All patients who suffer from cancer or other catastrophic illness/injury are issued a catastrophic illness/injury certificate (CIC), and these patients are exempted from copayment to the National Health Insurance (NHI) plan if they receive care for their catastrophic illness. The NHIRD contains comprehensive information on health care utilization of Taiwan National Health Insurance beneficiaries. The analyzed database of all claims was de-identified and all information of patients’ identifiers in NHIRD is double encrypted to protect patient confidentiality. Privacy policies were implemented to protect the privacy of beneficiaries, and all analyses were accomplished in the Health and Welfare Data Science Center (HWDC) of the Ministry of Health and Welfare, Taiwan. This study was approved by the Research Ethics Committee of China Medical University and Hospital (CMUH109-REC2-031(CR-3)). All the methods are in accordance with relevant guidelines and regulations.

### Cohort identification

We identified female patients with a diagnosis of hypothyroidism or hyperthyroidism (thyroid disease, TD), based on ICD-9-CM code 242 and ICD-10-CM codes from January 2000 to December 2018, as mentioned in Supplementary Table 1. Hyperthyroidism was defined as any hyperthyroid disease, including Grave’s disease, and hypothyroidism as any hypothyroid disease, including Hashimoto’s disease. Additional criteria were that patients had to receive the same diagnosis on at least 3 outpatient visits or 1 inpatient stay. Patients were only included if the first diagnosis of hyperthyroidism or hypothyroidism occurred before the date of first EC, UC or OC diagnosis. Patients with a diagnosis of thyroid disease were defined as the exposed group; patients who did not have a diagnosis of TD were defined as the non-exposed group (euthyroid group). Using encrypted patient identification numbers, the patient cohort was then linked to the catastrophic patient registry (HV) to identify newly diagnosed primary gynecological cancers—including endometrial cancer (EC), uterine corpus cancer (UC), and ovarian cancer (OC)—according to ICD codes (Supplementary Table 1). We did not included cervix uteri cancer because its incidence rate has already been declined dramatically after the National health insurance includes it in the four cancer screenings. For the outcomes of interest, we censored and followed the study cohort from the date of their first diagnosis of hypothyroidism or hyperthyroidism until the diagnosis of gynecologic cancers (EC, UC, or OC), death, or at the end of the follow-up time on December 31, 2018, whichever appeared first.

Patients were excluded if they had a history of postoperative hypothyroidism ICD-9 244.9, Thyroid malignancy: ICD9-193 before the first diagnosis of hyperthyroidism or hypothyroidism or if they had a diagnosis of hypothyroidism and hyperthyroidism on the same date; or they were diagnosed with hyperthyroidism and subsequently developed hypothyroidism or vice versa; or women less than 1 year follow-up and autoimmune thyroid diseases. The date of first diagnosis of thyroid disease was defined as the index date. We also excluded patients with a history of site-specific gynecologic cancers before the index date.

### Potential confounders

Potential confounding factors included patient demographic and clinical features, such as age, comorbidities (hyperlipidemia, obesity, hypertension, diabetes mellitus, ovarian dysfunction, and female infertility), co-medications (estrogen-containing oral, injectable, or topical products, or hormone replacement therapy), length of follow-up (years), which was categorized as < 6 years and > 6 years. We evaluated all factors in the year preceding cohort entry, except for co-medications, which were evaluated within the previous 6 months. The comorbidities identified in this study were strong potential risk factors for EC^4^. The Charlson Comorbidity Index (CCI) score is the most widely used tool to adjust for confounding due to comorbidities in epidemiological studies^23^. We thus used the CCI to adjust for comorbidities, with diseases defined by ICD-9-CM or ICD-10-CM codes. Comorbidities were only included if they were diagnosed at least twice during outpatient or hospital visits in order to increase the validity of comorbidity diagnoses in this study. Patients with the comorbidities in this study were classified into one of three categories according to the CCI. All confounders are listed in Table 1.

**Table 1:** Characteristics after propensity score matching among patients with hyperthyroidism and hypothyroidism.

Characteristics	Non-hyperthyroidism		Hyperthyroidism		p value	Characteristics	Non-hypothyroidism		Hypothyroidism		p value
	N = 296,872		N = 296,872				N = 44,852		N = 44,852		
	Patient number	%	Patient number	%			Patient number	%	Patient number	%	
Age					> 0.9999	Age					> 0.9999
20–40	129,156	43.51	129,160	43.51		20–40	10,463	23.33	10,467	23.34	
40–65	135,735	45.72	135,728	45.72		40–65	23,225	51.78	23,220	51.77	
65+	31,981	10.77	31,984	10.77		65 +	11,164	24.89	11,165	24.89	
Age, mean (sd)	44.07	15.19	44.06	15.14	0.7897	Age, mean (sd)	53.02	16.17	53.04	16.11	0.8264
Comorbidities						Comorbidities					
Hyperlipidemia	64,246	21.64	64,244	21.64	0.9950	Hyperlipidemia	12,846	28.64	12,842	28.63	0.9764
Obesity	5450	1.84	5464	1.84	0.8924	Obesity	1228	2.74	1221	2.72	0.8860
Hypertension	70,345	23.70	70,325	23.69	0.9513	Hypertension	13,420	29.92	13,419	29.92	0.9942
Diabetes mellitus	9335	3.14	9362	3.15	0.8410	Diabetes mellitus	4165	9.29	4160	9.27	0.9541
Ovarian dysfunction	10,632	3.58	10,642	3.58	0.9443	Ovarian dysfunction	1630	3.63	1636	3.65	0.9148
Female infertility	12,046	4.06	12,064	4.06	0.9058	Female infertility	1570	3.50	1573	3.51	0.9566
Medications						Medications					
Estrogen	111,753	37.64	130,052	43.81	< 0.0001	Estrogen	16,602	37.02	20,045	44.69	< 0.0001
Tamoxifen	4823	1.62	5442	1.83	< 0.0001	Tamoxifen	704	1.57	911	2.03	< 0.0001
Methimazole	–	–	133,594	45.00	–	Levothyroxine	–	–	30,977	69.06	–
Propylthiouracil	–	–	95,398	32.13	–						
Radioactive iodine	–	–	2978	1.00	–						
Thyroid surgery	–	–	20,352	6.86	–						
Charlson Comorbidity Index (CCI)					< 0.0001						0.0774
< 1	277,387	93.44	277,079	93.33		< 1	32,053	71.46	31,818	70.94	
1–3	14,630	4.93	15,118	5.09		1–3	3182	7.09	3139	7.00	
> 3	4855	1.64	4675	1.57		> 3	9617	21.44	9895	22.06	
Follow-up (year)					< 0.0001	Follow-up (year)					0.083
< 6	86,183	29.03	82,721	27.86		< 6	32,053	71.46	31,818	70.94	
6+	210,689	70.97	214,151	72.14		6+	12,799	28.54	13,034	29.06	

Remarks: *PY *person-year, *IR* incidence rate, per 1000 persons/years, *cHR* Crude Hazard ratio, *aHR* adjusted hazard ratio; significant p < 0.05.

### Propensity score model

Propensity score matching is often used to reduce selection bias in observational studies and used to minimize the confounding factor that frequently occurs in observational studies^24,25^. In our study, pairs were matched in 1:1 ratio based on their calculated propensity scores. Propensity scores were calculated from the results of a multivariate logistic regression model that included a set of confounders including age, sex, an index year at diagnosis, and CCI score category. One on one matches using the greedy algorithms. The algorithm makes "best" matches’ first and "next-best" matches next, in a hierarchical sequence until no more matches can be made. Best matches are those with the highest digit match on propensity score. The algorithm proceeds sequentially to the lowest digit match on propensity score (1 digit; 0.1). This will be called an 8 → 1 Digit Match. For the cases, the date of the index date as the date in which the cases received their first thyroid disease diagnosis. For controls, the date of the index date was defined as a random date within the study period.

### Statistical analysis

The chi-square test was used to compare the characteristics of women stratified by hyperthyroidism and hypothyroidism as categorical variables, and a t-test to compare groups stratified by continuous variables with a normal distribution. The Kaplan–Meier method was used to estimate the cumulative incidence of site-specific gynecologic cancers in women with or without hyperthyroidism or hypothyroidism, which were compared with the log-rank test. Logistic regression was used to calculate the propensity scores. Cox proportional hazards regression models were used to assess the effect of confounders on the risk of interested gynecological cancers in women with hyperthyroidism and hypothyroidism. Univarate analysis was performed first for each confounding factor, and then multivariate regression analysis was used to adjust the effect of confounding factors on the association of gynecologic cancer risk related to the hyperthyroidism and hypothyroidism. Confounding factors includes age, comorbidities, CCI score category, and medications. We also include a robust variance to account for within-subject correlation of individuals on the cox regression. All statistical analyses were carried out using SAS 9.4 (SAS Institute, Inc., Cary, NC, USA) and R studio (3.5.2). Differences were considered statistically significant if *p* < 0.05.

### Ethical approval

In Taiwan, ethical review is not required for National Health Insurance Research database. The researchers did not know the personal identity of the registered persons because the analyzed database of all claims was de-identified and all information of patients’ identifiers in NHIRD is double encrypted to protect patient confidentiality.

## Results

### Baseline characteristics

**Figure 1:**
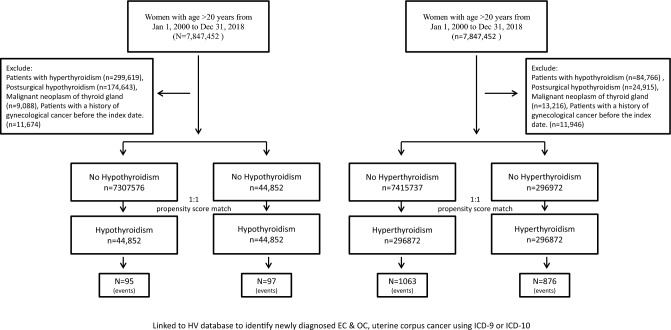
Flow chart of participants selection and study design.

After specifying exclusion criteria, a total of 15,065,037 women with thyroid disease aged ≥ 20 years were identified as eligible study cohorts. After 1:1 matching, a total of 296,872 hyperthyroid and 44,852 hypothyroid women were identified; 296,872 and 44,852 individuals without hyperthyroidism and hypothyroidism were matched (Fig. 1).The mean patient age was 44.07 ± 15.19 years in the non-hyperthyroidism group versus 44.06 ± 15.4 years in the hyperthyroidism group (*p* = 0.7897); and 53.02 ± 16.17 years in the non-hypothyroidism group versus 53.04 ± 16.11 years in the hypothyroidism group (*p* = 0.8264). Use of estrogen-containing medications (at any time) was recorded for 43.8% of the hypothyroidism group versus 37.64% of the non-hypothyroidism group (*p* < 0.0001), and by 44.69% of the hyperthyroidism group versus 37.02% of the non-hyperthyroidism group (*p* < 0.0001). Prior to the diagnosis of site-specific gynecological cancer, 2978 patients (1%) in the hyperthyroidism group had received radioactive iodine treatment (Table 1).

### Comparisons of incidence rates in interested gynecological cancers patients with or without thyroid diseases

**Figure 2:**
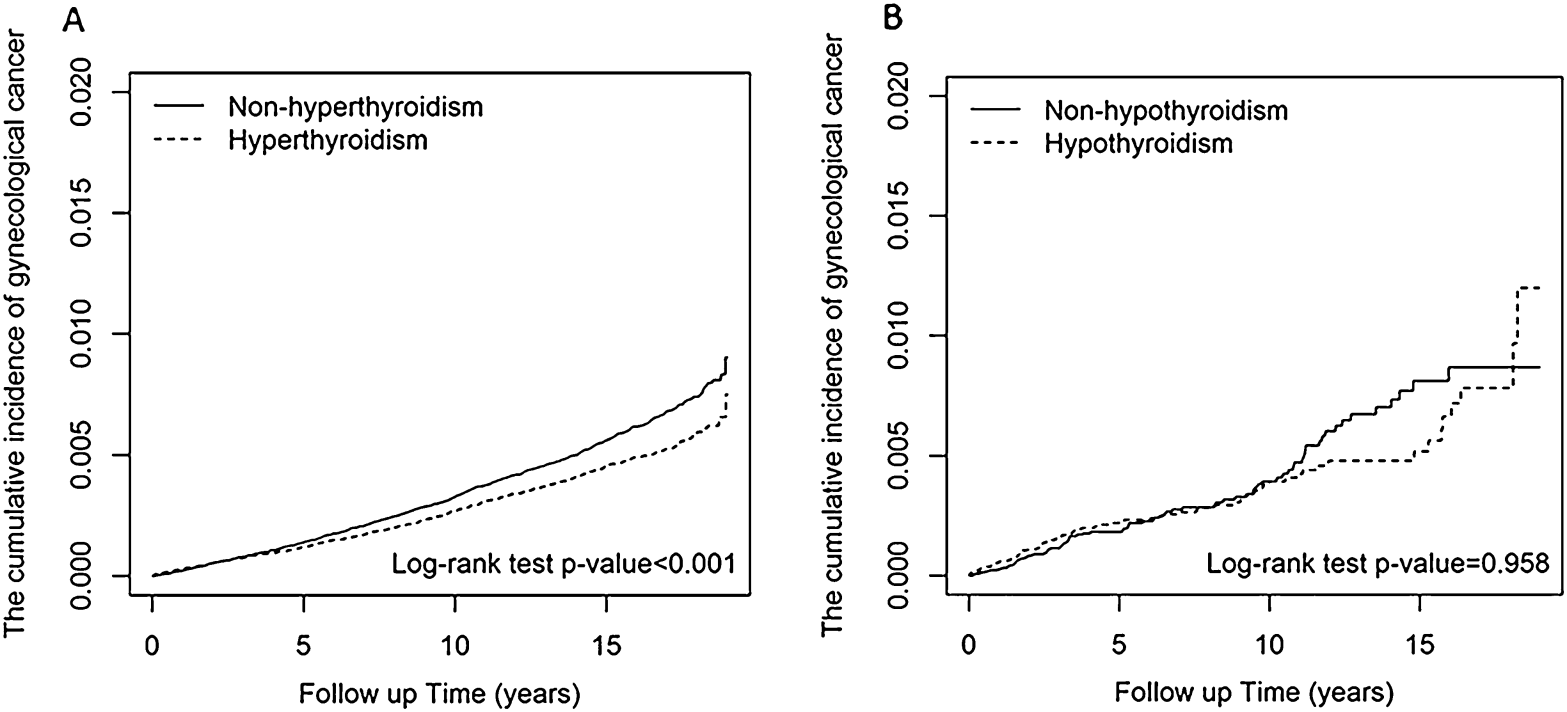
The cumulative incidence of gynecological cancer. (**A**) Hyperthyroidism, HT (dashed line) versus non-HT (solid line). (**B**) Hypothyroidism, HP (dashed line) versus non-HP (solid line).

Hyperthyroidism was associated with a significantly reduced risk and hypothyroidism showed slightly increased risk of gynecological cancers (aHR: 0.86; 95% CI 0.77, 0.96, < 0.0084) compared to women without hyperthyroidism and hypothyroidism, respectively (Table 2). Figure 2 showed that the hyperthyroidism group had a significantly lower cumulative incidence of gynecological cancer compared with the non-hyperthyroidism group (*p* < 0.001). In contrast, the cumulative incidence of gynecologic cancers did not significantly differ between the hypothyroidism and non-hypothyroidism groups. The hazard ratio and its 95% CI using robust variance were presented in the Supplementary Table 2. The results indicate that the risk patterns remain highly similar whether or not robust variance is included in the regression. Follow-up in the first 6 years, the hyperthyroidism and hypothyroidism group had 1.07-fold and 1.37-fold greater risk of developing gynecological cancers. After 6 years of follow-up, the incidence rate of interested gynecological cancers showed lower in the hyperthyroidism group than in the non-hyperthyroidism group (0.18 vs. 0.23/1000 person-years). Notably, the hyperthyroidism-associated risk reduction persisted beyond 6 years of follow-up (aHR: 0.75, 95% CI 0.65–0.87, p < 0.001) (Table 2).

**Table 2:** Incidence and adjusted confounders in hyperthyroidism and hypothyroidism patients related to gynecological cancers.

	Without hyperthyroidism			With hyperthyroidism			Adjusted HR^b^ (95% CI)		
	Event	PY	IR^a^	Event	PY	IR^a^	aHR	(95% CI)	p value
All Gyn cancers	1063	2,948,076	0.36	876	3,000,281	0.29	0.86	(0.77, 0.96)**	0.0084
Age, years									
20–40	308	1,399,103	0.22	228	1,406,870	0.16	0.72	(0.57, 0.90)**	0.0043
40–65	673	1,324,956	0.51	594	1,355,691	0.44	0.82	(0.55, 1.20)	0.3485
65+	82	224,017	0.57	54	237,720	0.45	0.94	(0.82, 1.07)	0.2987
Comorbidities									
Hyperlipidemia									
No	737	2,256,026	0.33	638	2,290,276	0.28		Reference	
Yes	326	692,050	0.47	238	710,005	0.34	0.70	(0.57, 0.86)***	< 0.001
Obesity									
No	1032	2,888,476	0.36	849	2,939,570	0.29		Reference	
Yes	31	59,600	0.52	27	60,711	0.44	0.81	(0.43, 1.54)	0.5269
Hypertension									
No	697	2,229,606	0.31	634	2,254,083	0.28		Reference	
Yes	366	718,471	0.51	242	746,198	0.32	0.67	(0.55, 0.83)***	< 0.001
Diabetes mellitus									
No	1040	2,892,830	0.36	860	2,939,660	0.29		Reference ara>	
Yes	23	55,246	0.42	16	60,621	0.26	0.56	(0.26, 1.24)	0.1532
Ovarian dysfunction									
No	1011	2,836,736	0.36	839	2,888,546	0.29		Reference	
Yes	52	111,340	0.47	37	111,735	0.33	1.01	(0.62, 1.62)	0.981
Female infertility									
No	1023	2,821,438	0.36	837	2,873,243	0.29		Reference	
Yes	40	126,638	0.32	39	127,038	0.31	1.22	(0.72, 2.06)	0.4549
Medications									
Estrogen									
No	642	1,796,340	0.36	480	1,629,601	0.29		Reference	
Yes	421	1,151,736	0.37	396	1,370,681	0.29	0.79	(0.66, 0.93)**	0.0047
Tamoxifen									
No	1055	2,899,031	0.36	873	2,941,733	0.30		Reference	
Yes	8	49,045	0.16	3	58,549	0.05	0.23	(0.03, 1.85)	0.1665
Follow-up									
< 6	451	288,198	1.56	388	277,154	1.40	1.07	(0.91, 1.27)	0.4007
6+	612	2,659,878	0.23	488	2,723,127	0.18	0.75	(0.65, 0.87)***	< 0.001

	Without hypothyroidism			With hypothyroidism			Adjusted HR (95% CI)^b^		
	Event	PY	IR	Event	PY	IR	aHR	(95% CI)	p value
All Gyn cancers	95	218,861	0.43	97	221,435	0.44	1.13	(0.74, 1.71)	0.5807
20–40	16	53,545	0.30	16	53,535	0.30	1.00	(0.50, 2.00)	0.9967
40–65	67	125,385	0.53	69	127,758	0.54	1.26	(0.76, 2.09)	0.3658
65+	12	39,931	0.30	12	40,141	0.30	1.38	(0.48, 3.98)	0.5489
Comorbidities									
Hyperlipidemia									
No	59	148,088	0.40	71	149,412	0.48		Reference	
Yes	36	70,773	0.51	26	72,023	0.36	0.68	(0.3, 1.53)	0.3488
Obesity									
No	87	211,772	0.41	93	214,298	0.43		Reference	
Yes	8	7089	1.13	4	7137	0.56	0.35	(0.04, 2.82)	0.3213
Hypertension									
No	56	147,236	0.38	66	148,074	0.45		Reference	
Yes	39	71,625	0.54	31	73,360	0.42	0.96	(0.48, 1.94)	0.9132
Diabetes mellitus									
No	83	204,261	0.41	91	205,693	0.44		Reference	
Yes	12	14,600	0.82	6	15,742	0.38	0.83	(0.23, 3.05)	0.7787
Ovarian dysfunction									
No	87	211,256	0.41	89	213,781	0.42		Reference	
Yes	8	7605	1.05	8	7654	1.05	1.01-	(0.76, 1.35)	0.9507
Medications									
Estrogen									
No	59	135,700	0.43	45	117,990	0.38		Reference	
Yes	36	83,160	0.43	52	103,445	0.50	1.2	(0.64, 2.22)	0.5699
Follow-up (years)									
< 6	59	76,689	0.77	69	75,456	0.91	1.37	(0.85, 2.20)	0.196
6+	36	142,172	0.25	28	145,979	0.19	0.49	(0.17, 1.37)	0.173

Remarks: *Gyn* gynecological cancer, *EC* endometrial cancer, *UC* uterine corpus cancer, *OC* ovary cancer. *PY* person-years.

p < 0.001. ^a^IR, incidence rate, rate per 1000 person-years. ^b^Adjusted HR: multivariable analysis including age, and comorbidities of diabetes, hypertension, hyperlipidemia, heart disease. ^#^Cox proportional hazards regression analyses for risk of gynecological cancers.

### Adjustment for confounders

We adjusted all the confounders using multivariate Cox proportional hazards regression models. We observed a significantly lower risk of interested gynecological cancers in the younger group (20–40 years) of patients with hyperthyroidism; this association was not found in other age groups. The increased risk of interested gynecological cancers in patients with hypothyroidism did not reach statistical significance in any of the three age groups (Table 3). Further adjusted analysis based on all other confounding factors revealed that dyslipidemia, hypertension were significantly associated with reduced risks of gynecological cancers among hyperthyroidism patients compared with patients without hyperthyroidism; while no statistically significant differences associated with any confounding factors among patients with hypothyroidism compared to patients without hypothyroidism (Table 2). However, a statistically significant reduction and a slightly increased risk of gynecologic cancer were found, respectively, in hyperthyroid and hypothyroid patients who had ever used estrogen.

In the subgroup analysis, we examined whether medication treatment for hyperthyroidism or hypothyroidism would change the risk of having gynecological cancers. Overall, no statistically significant risk of developing gynecological cancers was observed after adjustment of medications in patients with hyperthyroidism or hypothyroidism (HR = 0.99, 95% CI 0.87–1.13, p = 0.90; HR = 0.80, 95% CI 0.54–1.18, p = 0.262), respectively (Table 3). However, in women with hyperthyroidism, the HR increased to 1.32 and did not reach significance due to limited sample size. As sample size increases, one might expect statistical significance; therefore, caution is required in interpretation.

**Table 3:** Medication-adjusted subgroup analysis of hyperthyroidism and hypothyroidism associated with gynecological cancers.

Variables	Event	PY	IR	cHR	(95% CI)	p value	aHR	(95% CI)	p value
Hyperthyroidism									
Medications									
Endometrial cancer and uterine corpus cancer									
No	392	1,253,397	0.31	1.00	(reference)	–	1.00	(reference)	–
Yes	484	1,746,884	0.28	0.9	(0.79, 1.03)	0.1176	0.96	(0.84, 1.10)	0.5757
Ovarian cancer									
No	196	1,253,397	0.16	1	(reference)	–	1	(reference)	–
Yes	246	1,746,884	0.14	0.91	(0.75, 1.09)	0.3047	1.32	(0.89, 1.96)	0.1718
Overall							0.99	(0.87, 1.13)	0.900
Hypothyroidism									
Medications									
Endometrial cancer and uterine corpus cancer									
No	29	57,027	0.51	1.00	(reference)	–	1.00	(reference)	–
Yes	68	164,408	0.41	0.86	(0.56, 1.33)	0.4962	0.85	(0.53, 1.35)	0.4855
Ovarian cancer									
No	13	57,027	0.23	1	(reference)	–	1	(reference)	–
Yes	27	164,408	0.16	0.78	(0.4, 1.53)	0.4743	0.70	(0.34, 1.42)	0.317
Overall							0.80	(0.54, 1.19)	0.270

Remarks: gynecological cancer: endometrial cancer, uterine corpus cancer, ovary cancer; medications for hyperthyroidism patients included estrogen,

tamoxifen, methimazole, propylthiouracil and radioactive iodine; for hypothyroidism patients included estrogen, tamoxifen and levothyroxine;

Adjusted HR (hazard ratio) was adjusted for age, estrogen use and history of medication treatments by multivariable logistic regression analysis.

significant p < 0.05.

## Discussion

This is the first study specifically to assess the risk of interested gynecologic cancers associated with hyperthyroidism or hypothyroidism. We found that women with hyperthyroidism had a significantly lower risk of developing gynecologic cancers. On the other hand, women with hypothyroidism tended to have a slightly increased risk regardless of drug treatment. Age-stratified analyses revealed significantly reduced risk of gynecological cancer in patients with hyperthyroidism under the age of 40, but no significant association between age groups in patients with hypothyroidism.

The results of this large cohort study is likely to be supported by two previously published observational studies conducted by Chen et al., they reported an increased risk and decreased risk of EC in patients with hypothyroidism and hyperthyroidism, although this association was not statistically significant^26,27^. Their findings may also be supported by a recently published retrospective study in which an inverse correlation between TSH and FT4 was observed in a cancer patient population and may indirectly explain our findings^28^. Another large cohort study published previously by Kang et al. showed inconsistent results that hypothyroidism or hyperthyroidism were not significantly associated with EC or OC risk^29^. In addition, a previously published clinical study suggests that the risk of OC may significantly associate with hyperthyroidism^10^, but not associated with hypothyroidism^30^. The inconsistencies between study findings in this field remain unresolved. Differences between these studies may be due to the functional significant expression of thyroid hormones and receptors in different tumors. Under physiological conditions, thyroid hormone receptors control tumor cell proliferation, cancer cell defense pathway and produce tumor suppressor effects, nuclear thyroid hormone receptor-β1 (TRβ1) expression was detected in the normal epithelium (96%), but lower expression in adenomas (83%) and in cancer (68%) in colorectal mucosa and in colorectal tumors^31,32^. Another study indicated a tumor suppressor role of wild-type expressed TRβ1 in thyroid cancer and other cancer type, such as breast tumorigenesis^15,33^. On the contrary, abnormal expression or mutation of TRβ1 has been shown to promote carcinogenesis^15^.

Furthermore, we conducted an analysis of the follow-up time and results, dividing them into half of study period (6 years). Based on our follow-up time over 6 years, we found that women with hypothyroidism and hyperthyroidism had a lower risk of interested gynecological cancers. Our results are likely to be supported by a study conducted by Klaudia Żak et al.^34^. They found that patients with hyperthyroidism had a reduced risk of invasive ovarian cancer and prolonged survival (HR = 1.22 down to 1.07) after more than 5 years of follow-up. However, in patients with hypothyroidism, although the elevated hazard ratio (HR) did not reach statistical significance due to a limited sample size, an increased sample size could potentially lead to statistical significance.

In 2013, Seebacher et al. published the first study to report that baseline serum TSH > 2.5 mU/L was an independent prognostic parameter associated with poor disease survival, and with increased risk of EC recurrence. They also hypothesized that serum TSH measurements could be used as independent prognostic parameters of EC patient survival and their recurrence during EC follow-up^35^. However, no associations were found between elevated pre-therapeutic serum TSH levels, advanced FIGO (International Federation of Gynecology and Obstetrics) tumor stage and other risk factors in that study, some researcher suggested that TSH might be associated with systemic processes that interact with carcinogenesis (e.g., hormonal imbalances or inflammation)^9^. The specific mechanism by which serum TSH levels affect EC or OC risk remains unclear. This result may be explained that thyroid hormones are multifaceted function, including cellular growth, embryonic development, differentiation, metabolism proliferation, and the complicated modulation mechanism of thyroid hormones. Those are genomic and nongenomic actions^36^. These inconsistent research reports might reflect differences in tumor genesis between EC, OC and other types of cancer. In addition, EC, OC development in hypothyroidism patients may be influenced by many well-documented risk factors, such as age in elderly patients, obesity, hypertension, diabetes mellitus, postmenopausal estrogen replacement, ovarian dysfunction, infertility, nulliparity, and tamoxifen use^3,4,9^. Framework of unopposed estrogen hypothesis can explain most risk factors for EC. This hypothesis suggests that long-term exposure to estrogen increases the mitotic activity of endometrial cells and increases the number of DNA replication errors and somatic mutations, resulting in malignant phenotypes. Furthermore, the endometrium subsequently transforms to exhibit atypical hyperplasia and malignancy due to lack of progesterone-mediated differentiation^37,38^. This hypothesis could explain the results of this study on the trend toward increased risk of gynecological cancers following estrogen use in hypothyroid patients.

Another concern observed in our study was the age-adjusted analysis, which showed slightly increased risk of gynecological cancers in patients with hyperthyroidism and hypothyroidism beyond the age of 40. The result is consistent with recently published manuscript in 2023, they concluded that Individuals aged ≥ 60 years experienced the highest age-standardized prevalence rate of thyroid disease (15.4%; 95% CI 13.3–17.8%) and it may also be explained by advanced age is one of the top three risk factors for endometrial cancer reported in several articles^38,39^. Interestingly, we also observed that hyperthyroid patients with hyperlipidemia or hypertension may have a protective advantage on gynecological cancer risk. The results can be explained by the mechanism of thyroid hormones that regulate tissue and cellular metabolism. After the thyroid hormone receptors are activated by their ligands, it functions as a transcription factor. TH effects in the heart and brown adipose tissue are mediated by the THR isoform THR-α, whereas the THR isoform THR-β mediates the effects of TH on thyroid-stimulating hormone secretion and cholesterol metabolism. Mutations of the THR-β gene cause thyroid hormone resistance syndrome, characterized by tachycardia and increased TSH and free tetra-iodothyronine (FT4) levels. Hyperthyroidism has been shown to increase lipolysis in adipose tissue and lipogenesis in the liver. In particular, hyperthyroidism leads to increased catabolism resulting in weight loss, while hypothyroidism reduces the hepatic uptake of triglyceride-derived FFA, which is related to liver reduced lipolysis, fat tissue and cholesterol reduction^40,41^.

Overall, the mechanisms of thyroid hormone (TH) action are diverse and through a genomic and non-genomic pathway. The non-genomic pathway is the binding of thyroid hormones to integrin αvβ3 on the cell surface to activate the phosphatidylinositol-3-kinase (PI3K-Akt) and the mammalian target of rapamycin (mTOR) signaling pathways, and the mitogen-activated protein kinase/extracellular signal-related kinase 1/2 (MAPK/ERK 1/2) pathways, which stimulate cancer cell proliferation and angiogenesis. MAPK and PI3k are also stimulated by cytoplasmic TH receptor beta, which is probably associated with ovarian cancer aggressiveness^36^. The hypotheses can be proved in a study conducted by Shinderman-Mama et al. They found that thyroid hormones play a key role as potent αvβ3 ligands that promote ovarian cancer cell proliferation^42^. They thus used a αvβ3 blocker to reduce gene expression in order to disrupt the axis. This finding could be considered as a new therapeutic strategy for this aggressive disease^16,43^.

The genomic pathway is the binding of T3 to the thyroid hormone receptors (THRs) in the nucleus to exert its cellular effect^44^. The THRs proteins are encoded by the THRα and thyroid hormone receptor beta (THRB) genes^45^. THRB is a nuclear hormone receptor (NHR) that modulates growth signaling in both normal human development and cancerous tissue^46^. THRB expression is lost in a subset of endometrial carcinomas and is associated with poorer 5-year survival^45^.Sharma et al. reported that EC might be related to hypothyroidism^17^. The loss of expression of NHRs, particularly of ER and PR, has been associated with poor clinical outcomes in endometrial carcinoma. This assumption may be supported by a previous published study that THRB gene loss expression in women with endometrial cancer and integrin αvβ3 is overexpressed in ovarian cancer^45^.

Our study was able to minimize selection bias by limiting inclusion criteria to patients with at least 3 outpatient visits or 1 hospitalization with a definitive diagnosis of hyperthyroidism or hypothyroidism. The first weakness is the possibility of underestimation or overestimation because the study data were retrieved from administrative claims databases. Second, because lifestyle factors such as obesity and alcohol consumption that affect gynecologic cancer risk are not easily measured directly, the observed association between thyroid disease and gynecologic cancer risk may be underestimated. Third, laboratory data are not available in the National Health Insurance Research Database (NHIRD). We therefore cannot link TSH levels and thyroid hormone status at diagnosis to gynecological cancer risk.

## Conclusions

The main results of our study showed a significantly reduced risk of interested gynecological cancers associated with hyperthyroid women; while women with hypothyroidism trend towards increased risk of gynecologic cancers, although association is not statistically significant. This result may arouse oncologist and healthcare decision maker to alert the trend of gynecological cancer in Taiwan^2^. In addition to the health promotion efforts undertaken by the government so far^46^, clinical practitioners and basic researchers need to collaborate together investigating effective therapeutic strategies in a large scale study based on a clearer understanding of the mechanisms by which THs regulate reproductive function through thyroid hormone receptors-mediated genomic and integrin receptor-associated non-genomic pathways^35^.

### Supplementary Information

Supplementary Information 1.

Supplementary Information 2.

## Data Availability

All data generated or analyzed during this study are included in this published article and its submitted supplementary information files.
