# Supplementary material for: Hypothyroidism and hyperthyroidism related to gynecologic cancers: a nationwide population-based cohort study
Source: Sci Rep. 2024 Jan 22;14:1892. doi: 10.1038/s41598-023-50439-z (PMC10803809; doi:10.1038/s41598-023-50439-z)
Supplement: Supplementary file 1 — Supplementary Information 1. [file 41598_2023_50439_MOESM1_ESM.docx]

**Supplementary Material**

**Supplementary Table 1. International Classification of Disease (ICD) codes**

| Endometrial cancer, EC: ICD-10: C54.1 |
| --- |
| Uterine corpus cancer: ICD-9: 179; 182.0-1; 182.8; ICD-10-CM: C55 |
| Ovarian cancer, OC: ICD-9: 183.0-183.9; 236.2; ICD-10: C56, C56.1, C56.2, C56.9 |
| Congenital hypothyroidism:  ICD-9: 243-244; ICD-10: E00-E89; Unspecified acquired hypothyroidism: ICD-9 244.9, E03.9 |
| Hyperthyroidism: ICD-9: 242.01-242. 9, ICD-10: E05-E05.9,ICD-10: E05.0B |
| **Charlson Comorbidity Index : Co-morbidities( risk factors)** |
| **1 score**  Myocardial infarction  ICD-9: 410; ICD-10: I21, I22, I23  Congestive heart failure  ICD-9: 427.09, 427.10, 427.11, 427.19, 428.99, 782.49; ICD-10: I50, I11.0, I13.0, I13.2  Peripheral vascular disease  ICD-9: 440, 441, 442, 443, 444, 445; ICD-10: I70, I71, I72, I73, I74, I77  Hypertension:  ICD-9: 401.9; ICD-10: I10  Cerebrovascular disease  ICD-9: 430-438; ICD-10: I60-I69, G45, G46  Dementia  ICD-9: 290.09-290.19, 293.09; ICD-10: F00-F03, F05.1, G30  Chronic pulmonary disease  ICD-9: 490-493, 515-518, ICD-10: J40-J47, J60-J67, J68.4, J70.1, J70.3, J84.1, J92.0, J96.1, J98.2, J98.3  Connective tissue disease  ICD-9: 712, 716, 734, 446, 135.99; ICD-10: M05, M06, M08, M09, M30, M31, M32, M33, M34, M35, M36, D86  Ulcer disease  ICD-9: 530.91, 530.98, 531-534; ICD-10: K22.1, K25-K28  Mild liver disease  ICD-9: 571, 573.01, 573.04; ICD-10: B18, K70.0-K70.3, K70.9, K71, K73, K74, K76.0  Diabetes types 1 and 2  ICD-9: 249.00, 249.06, 249.07, 249.09, 250.00, 250.06, 250.07, 250.09; ICD-10: E10.0, E10.1, E10.9, E11.0, E11.1, E11.9  Dyslipidemia:  Ovarian dysfunction:ICD-10: E 28.0-28.9  Infertility: ICD-10: N46.8-46.9 ; ICD-10: N97.0-97.9 |
| **2 score**  Hemiplegia  ICD-9: 344; ICD-10: G81, G82  Moderate to severe renal disease  ICD-9: 403, 404, 580-583, 584, 590.09, 593.19, 753.10-753.19, 792; ICD-10: 12, I13, N00-N05, N07, N11, N14, N17-N19, Q61  Diabetes with end-organ damage  ICD-9: 249.01-249.05, 249.08, 250.01-250.05, 250.08; ICD-10: E10.2-E10.8, E11.2-E11.8  Any tumor ICD-9: 140-194; ICD-10: C75-C75.9  Leukemia  ICD-9: 204-207; ICD-10: C91-C95  Lymphoma  ICD-9: 200-203, 275.59; ICD-10: C81-C85, C88, C90, C96  Breast cancer: ICD-9: 170, ICD-10: C50  Prostate cancer: ICD-9: 185 , 233.4 , 222.2; ICD-10-CM: C61  Bladder cancer: ICD-9: 188.9; ICD-10-CM C67.9  Lung/ bronchus: ICD-9 :162.9  Colon: ICD-9-CM 153.9; ICD-10-CM C18.9  Rectal: ICD-9-CM 154.0, 154.1 and 154.8; ICD-10 C20  Pancreas: ICD-9-CM 157.0-157.9  Kidney/Renal: ICD-9-CM 189.0; ICD-10-CM C64.9 |
| Diabetes with end-organ damage  ICD-9: 249.01-249.05, 249.08, 250.01-250.05, 250.08; ICD-10: E10.2-E10.8, E11.2-E11.8 |
| **3 score** |
| Moderate to severe liver disease  ICD-9: 070.00, 070.02, 070.04, 070.06, 070.08, 573.00, 456.00-456.09;  ICD-10: B15.0, B16.0, B16.2, B19.0, K70.4, K72, K76.6, I85 |
| **6 score** |
| Metastatic solid tumor  ICD-8: 195-198, 199; ICD-10: C76-C80  AIDS  ICD-8: 079.83; ICD-10: B21-B24 |
| **Obesity**: ICD-9: 277.99. ICD-10: E66.0-66.9 |
| **Alcohol-related disease**  ICD-9: 291.00–291.99,303.00–303.99, 571.09, 571.10, 577.10, E861, N979, N980; ICD-10: E24.4, E52.9A, F10.0, F10.1, F10.2–10.9, G31.2, G62.1,G72.1, I42.6, K29.2, K70, K85.2, K86.0, T50.0A, T51, R78, Z50.2, Z71.4, Z72.1. |
