# Supplementary material for: Hypothyroidism and hyperthyroidism related to gynecologic cancers: a nationwide population-based cohort study
Source: Sci Rep. 2024 Jan 22;14:1892. doi: 10.1038/s41598-023-50439-z (PMC10803809; doi:10.1038/s41598-023-50439-z)
Supplement: Supplementary file 2 — Supplementary Information 2. [file 41598_2023_50439_MOESM2_ESM.pdf]

**Supplementary Table. Cox proportional hazards regression models were used to estimate the risk of gynecological cancers with a robust variance to account for within-subject correlation of individuals.**

| | Without Hyperthyroidism | | | With Hyperthyroidism | | | Univariate | | | Multivariate | | |
|---|---|---|---|---|---|---|---|---|---|---|---|---|
| **Variables** | **Event** | **PY** | **IR** | **Event** | **PY** | **IR** | **cHR** | **(95% CI)** | **p-value** | **aHR** | **(95% CI)** | **p-value** |
| **Overall** | 1063 | 2948076 | 0.36 | 876 | 3000281 | 0.29 | 0.81 | (0.74, 0.8 | <0.001 | 0.86 | (0.77, 0.9 | 0.0084 |
| | **Without hypothyroidism** | | | **With Hypothyroidism** | | | **Univariate** | | | **Multivariate** | | |
| **Variables** | **Event** | **PY** | **IR** | **Event** | **PY** | **IR** | **cHR** | **(95% CI)** | **p-value** | **aHR** | **(95% CI)** | **p-value** |
| **Overall** | 95 | 218861 | 0.43 | 97 | 221435 | 0.44 | 1.01 | (0.76, 1.3 | 0.9585 | 1.13 | (0.74, 1.7 | 0.5838 |
